# Crystal structure of the tetra­gonal polymorph of bis­(1-ethyl-3-methyl­imidazolium) tetra­bromido­cadmate

**DOI:** 10.1107/S2056989016009919

**Published:** 2016-06-24

**Authors:** Tamara Đorđević, Sabrina Gerger, Ljiljana Karanović

**Affiliations:** a Institut für Mineralogie und Kristallographie, Universität Wien, Althanstrasse 14, A-1090 Vienna, Austria; b Laboratory of Crystallography, Faculty of Mining and Geology, Đušina 7, 11000 Belgrade, Serbia

**Keywords:** crystal structure, 1-ethyl-3-methyl imidazolium bromide, ionothermal synthesis, tetra­bromido­cadmate, supra­molecular organization

## Abstract

Both unique Cd atoms in the tetra­gonal polymorph of bis­(1-ethyl-3-methyl­imidazolium) tetra­bromido­cadmate, (C_6_H_11_N_2_)_2_[CdBr_4_], occupy special positions (site symmetry -4). The crystal structure consists of isolated tetra­hedral [CdBr_4_]^2−^ anions which are surrounded by 1-ethyl-3-methyl­imidazolium cations. The methyl and ethyl side chains of the cations show positional disorder in a 0.590 (11):0.410 (11) ratio. In the crystal, (C_6_H_11_N_2_)^+^ cations display three weak C—H⋯Br hydrogen-bond inter­actions through the imidazolium ring H atoms with the Br^−^ ligands of the surrounding complex anions. The alkyl groups of the side chains are not involved in hydrogen bonding.

## Abstract 2

The title structure represents the tetra­gonal polymorph (the other known structure being monoclinic) and is isotypic with its [*M*Br_4_] analogues (*M* = Co, Ni, Zn).

## Chemical context   

Laboratories around the world have used ionic liquids to prepare many different types of solids, ranging from nanoparticles of different types, to semiconductors, and inorganic and organic solids (Morris, 2009 ▸). In an attempted synthesis of mineral-related arsenates, the ionic liquid 1-ethyl-3-methyl­imidazolium bromide (eminBr), C_6_H_11_BrN_2_, was tested as a solvent and template. C_6_H_11_BrN_2_ has a wide liquid range (despite being a solid at room temperature, with a melting point of 356 K), low vapour pressure and has been used extensively for ionothermal synthesis because it is a relatively polar solvent.
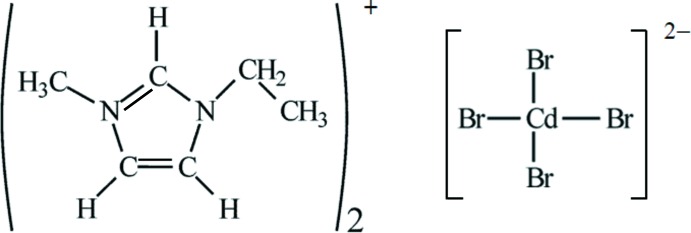


**Figure 1:**
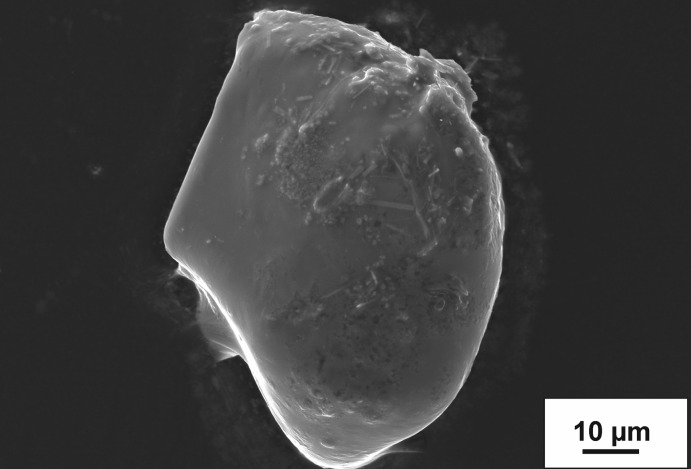
Back-scattered scanning electromicrograph of leaf-like (C_6_H_11_N_2_)_2_[CdBr_4_]. The small needle-like crystals on the top are from an unidentified Cd/Mn arsenate.

The title compound, (C_6_H_11_N_2_)_2_[CdBr_4_], was obtained under ionothermal conditions using eminBr as the solvate. The SEM–EDS study of the title compound showed small amounts of a cadmium–manganese arsenate in the form of small needle-like crystals up to maximal 15 µm on the top of the plate-like crystals of the title compound (Fig. 1 ▸). This phase is present in very small amounts and therefore could not be identified using powder or single-crystal X-ray diffraction techniques. The powder pattern indicated the tetra­gonal polymorph of the title compound as the main phase and the monoclinic polymorph (Gou *et al.*, 2016 ▸) as a minority phase.

## Structural commentary   

**Figure 2:**
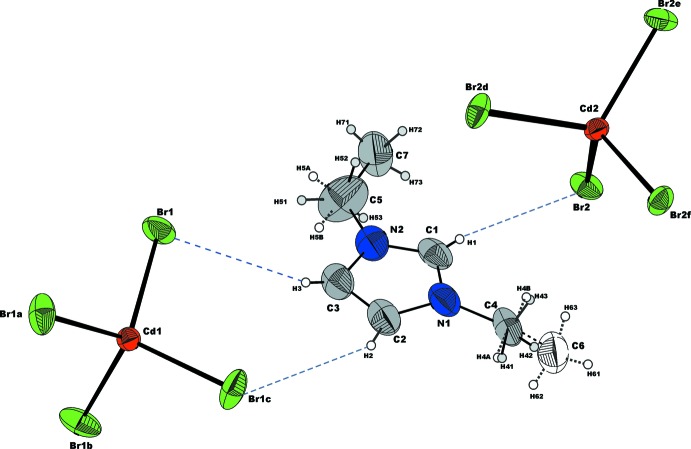
A view of the mol­ecular entities in the structure of (C_6_H_11_N_2_)_2_[CdBr_4_]. Displacement ellipsoids are drawn at the 50% probability level. H atoms are represented as small spheres of arbitrary radius. C—H⋯Br hydrogen-bonding inter­actions are shown with dashed blue lines. Disordered alkyl groups are distinguished by solid and dotted bonds, together with the C and H atoms being shown in different colours. [Symmetry codes: (*a*) *y* − 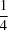, −*x* + 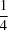, −*z* + 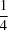; (*b*) −*y* + 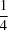, *x* + 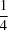, −*z* + 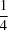; (*c*) −*x*, −*y* + 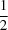, *z*; (*d*) −*y* + 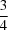, *x* + 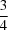, −*z* + 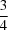; (*e*) *y* − 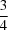, −*x* + 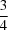, −*z* + 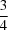; (*f*) −*x*, −*y* + 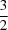, *z*.]

Emim, C_6_H_11_N_2_^+^, cations together with [CdBr_4_]^2−^ anions as discrete tetra­hedra are the main structural building units (Fig. 2 ▸). The imidazolium ring is, as expected, a planar, slightly distorted penta­gon. The deviation of the ring atoms from the least-squares plane is smaller than 0.006 (7) Å. The bond lengths of 1.356 (8) and 1.297 (7) Å for the N1—C1 and C1—N2 bonds, respectively, indicate conjugated double-bond character, having one bond slightly longer than the usual C=N double-bond length, 1.27 Å. The N1—C2 and N2—C3 bond lengths [1.360 (7) and 1.359 (8) Å] are shorter than a typical C—N single bond (1.472 ± 6 Å) and close to the shortened (partial double bond) in heterocyclic systems, 1.352 ± 5 Å, while the bond length of 1.373 (9) Å for C2—C3 is slightly longer than a typical C=C double bond of 1.337 ± 6 Å (Macgillavry & Rieck, 1968 ▸). The alkyl groups of the side chains showed strong anisotropic atomic displacements during refinement, suggesting a statistical positional disorder that was taken into account for the final model (Fig. 2 ▸). The carbon atoms C4, C5, C6 and C7 from the disordered alkyl groups of side chains are also planar and the largest deviation from the least-squares plane through the imidazolium ring atoms is 0.163 (16) Å for C7 and −0.949 (19) Å for C6, while C5 and C4 are just −0.013 (1) and 0.039 (1) Å, respectively, out of plane.

Both unique Cd atoms occupy special positions (on a fourfold rotoinversion axis parallel to the *c* axis, site symmetry 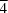). Consequently both tetra­bromido­cadmate anions possess crystallographically imposed 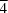 symmetry and therefore, each Cd atom bonds to four symmetry-related Br atoms (Fig. 2 ▸). The Cd1—Br1 bond length of 2.5745 (6) Å in the almost regular tetra­hedral configuration of the [Cd1Br_4_]^2−^ anion is slightly shorter than 2.5806 (5) Å for the [Cd2Br_4_]^2−^ anion. The Br—Cd—Br bond angles are 109.14 (3) and 109.64 (2)° in [Cd1Br_4_]^2−^ but 107.88 (1) and 112.71 (3)° in the slightly more distorted [Cd2Br_4_]^2–^ anion. The angular range for both anions is comparable with those reported by Sharma *et al.* (2006 ▸).

## Infrared spectroscopy   

Fourier-transform infrared (FT–IR) absorption single-crystal infrared spectra were recorded on a Bruker Tensor 27 FT–IR spectrophotometer with a mid-IR glowbar light source and KBr beam splitter, attached to a Hyperion2000 FT–IR microscope with a liquid nitro­gen-cooled mid-IR broad band MCT detector. A total of 128 scans were accumulated between 4000 and 550 cm^−1^ using a circular sample aperture (100 µm diameter) and ATR 15 × objective.

**Figure 3:**
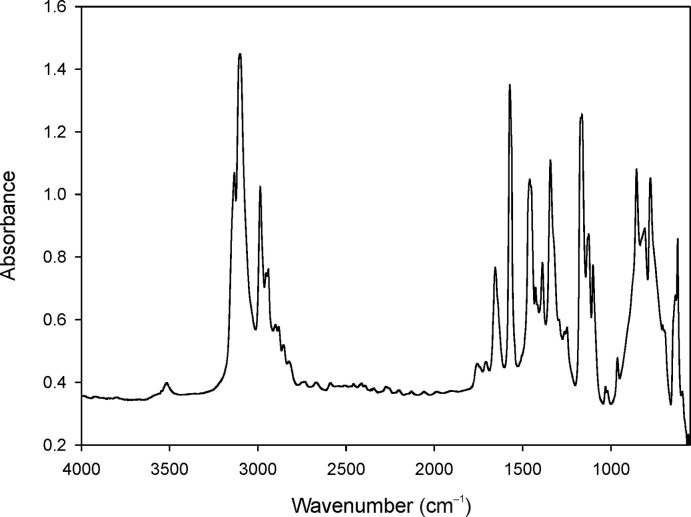
FT–IR spectrum of (C_6_H_11_N_2_)_2_[CdBr_4_].

The title compound shows characteristic bands of the imidazolium ring and the alkyl chains (Barbara, 2004 ▸; Nakamoto, 1978 ▸) (Fig. 3 ▸). The bands at 3134 and 3101 cm^−1^ can be attributed to aromatic C—H stretching (Tait & Osteryoung, 1984 ▸). Their relatively low values confirm the presence of weak hydrogen bonds. A higher wave number would indicate a diminution or absence of hydrogen bonds (Larsen *et al.*, 2000 ▸). The band at 2985 cm^−1^ can be attributed to aliphatic C—H stretching (Tait & Osteryoung, 1984 ▸); aliphatic C—H bending vibrations [δ(CH_2_), δ(CH_3_), δ_as_(CH_3_)] are located between 1470 and 1380 cm^−1^ (Katsyuba *et al.*, 2004 ▸) and mostly represented by the band at 1460 cm^−1^. The band at 1578 cm^−1^ is assigned to the C=C and C—N stretching vibrations of the imidazolium ring. Bands centred at 1342 and 1162 cm^−1^, respectively, represent the stretching vibrations between the alkyl chains and N atoms (Katsyuba *et al.*, 2004 ▸). All bands below 850 cm^−1^ can be attributed to the out-of-plane vibrations of the imidazolium cation (Katsyuba *et al.*, 2004 ▸). The most intense bands are located at 854, 775 and 621 cm^−1^. Even if there is no water in the structure of (C_6_H_11_N_2_)_2_[CdBr_4_], O—H vibrations may still be present because of the hygroscopic character of the ionic liquid.

## Supra­molecular features   

**Figure 5:**
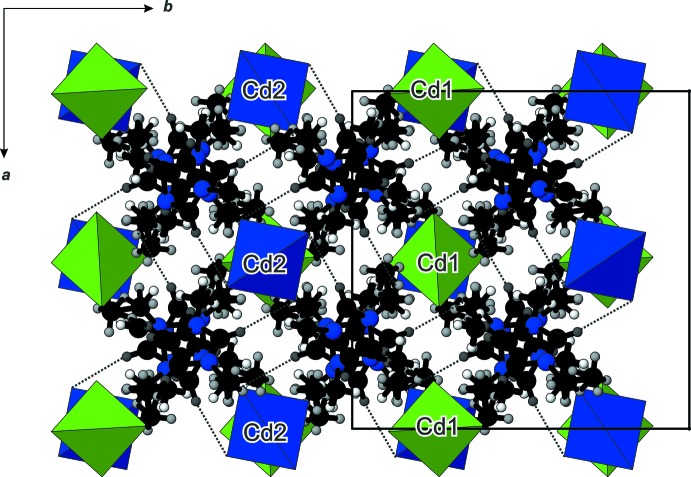
The projection of the structure of (C_6_H_11_N_2_)_2_[CdBr_4_], viewed down the *c* axis, normal to the channels formed by the supra­molecular organization of the imidazolium cations.

There are no significant inter­actions between [Cd2Br_4_]^2–^ anions, except a short Br1⋯Br1 contact which amounts to 3.764 (2) Å. The crystal packing of the cations and anions in a three-dimensional network is realized through C—H⋯Br inter­actions (Figs. 2 ▸ and 4 ▸, Table 1 ▸) involving the imidazolium ring H atoms (H1, H2 and H3), but not the H atoms of the alkyl side chains. Larsen *et al.* (2000 ▸) found that the imidazolium cation is often disordered whereby the disorder can take many different forms. They also have found that positional disorder of the cations in their crystal structures is a direct indicator of packing inefficiency, *i.e*. packing inefficiency becomes reflected in disorder when cation/anion inter­actions are reduced essentially to the level of van der Waals or very weak hydrogen-bonding-type forces. The resulting network in the title structure has a channel structure defined by the organization of the imidazolium cations, with the [CdBr_4_]^2–^ anions residing in the channels (Fig. 5 ▸).

## Database survey   

Tetragonal (C_6_H_11_N_2_)_2_[CdBr_4_] is isotypic with (C_6_H_11_N_2_)_2_[CoBr_4_] and (C_6_H_11_N_2_)_2_[NiBr_4_] (Hitchcock *et al.*, 1993 ▸), as well as (C_6_H_11_N_2_)_2_[ZnBr_4_] (Zhou *et al.*, 2010 ▸; Zhang & Liu, 2012 ▸). However, these three structures do not show any disorder of the imidazolium cations. The crystal structure of the monoclinic (C_6_H_11_N_2_)_2_[CdBr_4_] polymorph has also been reported recently (Gou *et al.*, 2016 ▸).

## Synthesis and crystallization   

A 1 g mixture of CdO, Mn(NO_3_)_2_·H_2_O, As_2_O_5_ in the molar ratio 2:2:1 was mixed with 2 g of molten emimBr and placed in a teflon container into a steel autoclave. A heating regime with three steps was chosen: the autoclaves were heated from 293 to 493 K (four h), held at 493 K for 72 h, and finally cooled to room temperature within 99 h. The obtained products were washed with ethanol, filtered and dried in the air at room temperature. The title compound crystallized as leafy-like crystals (yield *ca* 85%) together with crystals of the monoclinic polymorph (yield *ca* 10%) and small amounts of a yet unidentified Cd/Mn-arsenate (single-crystal size 10 µm). The crystals of tetra­gonal (C_6_H_11_N_2_)_2_[CdBr_4_]) are no longer than 0.15 mm in length.

## Refinement   

Crystal data, data collection and structure refinement details are summarized in Table 2 ▸. The imidazolium cation was modelled as disordered having approximate twofold rotation symmetry. The two orientations of the disordered cation are related to each other by a 180° rotation around the pseudo-twofold symmetry axis lying in the ring plane, connecting the C1 and bis­ecting the opposite C2—C3 bonds in the imidazolium ring. This causes a positional disorder of the methyl and ethyl side chains, with a site occupation ratio of 0.590 (11):0.410 (11). All hydrogen atoms attached to C atoms were placed in geometrically calculated positions and refined using a riding model, with C—H = 0.96 Å and *U*_iso_(H) = 1.5*U*_eq_(C) for methyl H atoms, C—H = 0.97 Å and *U*_iso_(H) = 1.2*U*_eq_(C) for methyl­ene H atoms, and C—H = 0.93 Å and *U*_iso_(H) = 1.2*U*_eq_(C) for imidazolium ring H atoms.

## Supplementary Material

Crystal structure: contains datablock(s) I. DOI: 10.1107/S2056989016009919/wm5286sup1.cif

Structure factors: contains datablock(s) I. DOI: 10.1107/S2056989016009919/wm5286Isup2.hkl

CCDC reference: 1486568

Additional supporting information: crystallographic information; 3D view; checkCIF report

## Figures and Tables

**Figure 4:**
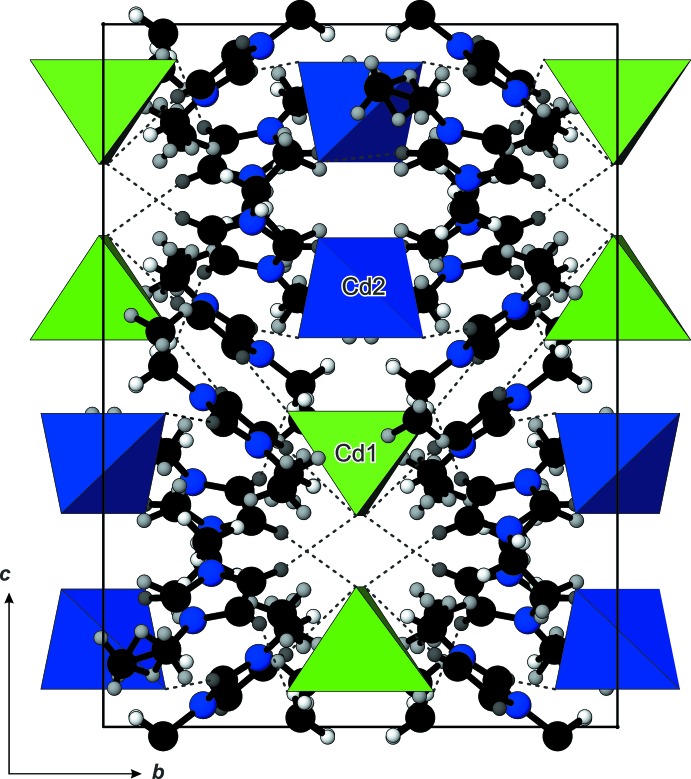
The packing of the structure of (C_6_H_11_N_2_)_2_[CdBr_4_], viewed down the *a* axis, showing the tetra­hedral [CdBr_4_]^2−^ anions linked to the emim, [C_6_H_11_N_2_]^+^, cations by hydrogen-bonding inter­actions. C and N atoms are presented as black and blue spheres, respectively, and H atoms as grey small spheres.

**Table 1:** Hydrogen-bond geometry (Å, °)

*D*—H⋯*A*	*D*—H	H⋯*A*	*D*⋯*A*	*D*—H⋯*A*
C1—H1⋯Br2^i^	0.93	2.77	3.679 (6)	167
C2—H2⋯Br1^ii^	0.93	2.93	3.824 (7)	161
C3—H3⋯Br1	0.93	2.90	3.753 (6)	154

Symmetry codes: (i) 
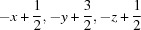
; (ii) 
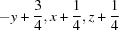
.

**Table 2:** Experimental details

Crystal data	
Chemical formula	(C_6_H_11_N_2_)_2_[CdBr_4_]
*M*_r_	654.38
Crystal system, space group	Tetragonal, *I*4_1_/*a*
Temperature (K)	100
*a*, *c* (Å)	14.691 (2), 20.075 (4)
*V* (Å^3^)	4332.8 (12)
*Z*	8
Radiation type	Mo *K*α
μ (mm^−1^)	8.39
Crystal size (mm)	0.15 × 0.02 × 0.01

Data collection	
Diffractometer	Stoe StadiVari with pixel array detector
Absorption correction	Multi-scan (*X-AREA* and *X-RED32*; Stoe, 2013 ▸)
*T*_min_, *T*_max_	0.366, 0.921
No. of measured, independent and observed [*I* > 2σ(*I*)] reflections	34206, 3016, 2046
*R*_int_	0.102
(sin θ/λ)_max_ (Å^−1^)	0.694

Refinement	
*R*[*F* ^2^ > 2σ(*F* ^2^)], *wR*(*F* ^2^), *S*	0.037, 0.074, 0.96
No. of reflections	3016
No. of parameters	94
No. of restraints	17
H-atom treatment	H-atom parameters constrained
Δρ_max_, Δρ_min_ (e Å^−3^)	0.89, −0.86

Computer programs: *X-AREA* and *X-RED32* (Stoe, 2013 ▸), *SIR97* (Altomare *et al.*, 1999 ▸), *SHELXL2014* (Sheldrick, 2015 ▸), *WinGX* (Farrugia, 2012 ▸), *ATOMS* (Dowty, 2000 ▸) and *publCIF* (Westrip, 2010 ▸).

## supplementary crystallographic information

### Crystal data

**Table:**

(C_6_H_11_N_2_)_2_[CdBr_4_]^−^	*D*_x_ = 2.006 Mg m^−^^3^
*M**_r_* = 654.38	Mo *K*α radiation, λ = 0.71073 Å
Tetragonal, *I*4_1_/*a*	Cell parameters from 18051 reflections
*a* = 14.691 (2) Å	θ = 5.6–63.4°
*c* = 20.075 (4) Å	µ = 8.39 mm^−^^1^
*V* = 4332.8 (12) Å^3^	*T* = 100 K
*Z* = 8	Leaf-like, colourless
*F*(000) = 2480	0.15 × 0.02 × 0.01 mm

### Data collection

**Table:**

Stoe StadiVari with pixel array detector diffractometer	3016 independent reflections
Radiation source: IµS microfocus source	2046 reflections with *I* > 2σ(*I*)
Plane graphite monochromator	*R*_int_ = 0.102
φ and ω scans	θ_max_ = 29.6°, θ_min_ = 2.8°
Absorption correction: multi-scan (*X-AREA* and *X-RED*; Stoe, 2013)	*h* = −17→20
*T*_min_ = 0.366, *T*_max_ = 0.921	*k* = −12→20
34206 measured reflections	*l* = −27→27

### Refinement

**Table:**

Refinement on *F*^2^	Primary atom site location: structure-invariant direct methods
Least-squares matrix: full	Secondary atom site location: difference Fourier map
*R*[*F*^2^ > 2σ(*F*^2^)] = 0.037	Hydrogen site location: inferred from neighbouring sites
*wR*(*F*^2^) = 0.074	H-atom parameters constrained
*S* = 0.96	*w* = 1/[σ^2^(*F*_o_^2^) + (0.0344*P*)^2^] where *P* = (*F*_o_^2^ + 2*F*_c_^2^)/3
3016 reflections	(Δ/σ)_max_ = 0.001
94 parameters	Δρ_max_ = 0.89 e Å^−^^3^
17 restraints	Δρ_min_ = −0.86 e Å^−^^3^

### Special details

**Table:**

Geometry. All esds (except the esd in the dihedral angle between two l.s. planes) are estimated using the full covariance matrix. The cell esds are taken into account individually in the estimation of esds in distances, angles and torsion angles; correlations between esds in cell parameters are only used when they are defined by crystal symmetry. An approximate (isotropic) treatment of cell esds is used for estimating esds involving l.s. planes.
Refinement. Refinement of F^2^ against ALL reflections. The weighted R-factor wR and goodness of fit S are based on F^2^, conventional R-factors R are based on F, with F set to zero for negative F^2^. The threshold expression of F^2^ > 2sigma(F^2^) is used only for calculating R-factors(gt) etc. and is not relevant to the choice of reflections for refinement. R-factors based on F^2^ are statistically about twice as large as those based on F, and R- factors based on ALL data will be even larger.

### Fractional atomic coordinates and isotropic or equivalent isotropic displacement parameters (Å^2^)

**Table:**

	*x*	*y*	*z*	*U*_iso_*/*U*_eq_	Occ. (<1)
Cd1	0.0000	0.2500	0.1250	0.02409 (13)	
Cd2	0.0000	0.7500	0.3750	0.02503 (13)	
Br1	0.00834 (3)	0.39254 (4)	0.05065 (3)	0.04833 (17)	
Br2	0.12129 (4)	0.66831 (3)	0.30377 (3)	0.03715 (13)	
N1	0.3058 (3)	0.5397 (3)	0.2191 (3)	0.0513 (12)	
N2	0.1979 (3)	0.5755 (3)	0.1517 (3)	0.0505 (12)	
C1	0.2669 (4)	0.6082 (4)	0.1840 (3)	0.0464 (13)	
H1	0.2864	0.6685	0.1832	0.056*	
C2	0.2585 (4)	0.4620 (4)	0.2067 (3)	0.0526 (14)	
H2	0.2708	0.4044	0.2237	0.063*	
C3	0.1891 (4)	0.4850 (4)	0.1641 (3)	0.0501 (14)	
H3	0.1447	0.4463	0.1471	0.060*	
C4	0.38352 (8)	0.54777 (5)	0.26324 (6)	0.073 (2)	
H41	0.3957	0.4900	0.2839	0.109*	0.590 (11)
H42	0.3710	0.5923	0.2970	0.109*	0.590 (11)
H43	0.4362	0.5664	0.2381	0.109*	0.590 (11)
H4A	0.3912	0.4890	0.2845	0.087*	0.410 (11)
H4B	0.4364	0.5574	0.2355	0.087*	0.410 (11)
C5	0.13928 (7)	0.62737 (8)	0.11001 (5)	0.090 (3)	
H51	0.0914	0.5888	0.0926	0.136*	0.410 (11)
H52	0.1721	0.6536	0.0738	0.136*	0.410 (11)
H53	0.1110	0.6756	0.1357	0.136*	0.410 (11)
H5A	0.1300	0.5909	0.0703	0.109*	0.590 (11)
H5B	0.0821	0.6312	0.1324	0.109*	0.590 (11)
C6	0.3888 (12)	0.6105 (11)	0.3126 (9)	0.066	0.410 (11)
H61	0.4458	0.6040	0.3356	0.099*	0.410 (11)
H62	0.3396	0.6016	0.3433	0.099*	0.410 (11)
H63	0.3849	0.6705	0.2938	0.099*	0.410 (11)
C7	0.1525 (10)	0.7140 (9)	0.0898 (8)	0.085	0.590 (11)
H71	0.1101	0.7285	0.0550	0.127*	0.590 (11)
H72	0.2135	0.7206	0.0733	0.127*	0.590 (11)
H73	0.1434	0.7546	0.1267	0.127*	0.590 (11)

### Atomic displacement parameters (Å^2^)

**Table:**

	*U*^11^	*U*^22^	*U*^33^	*U*^12^	*U*^13^	*U*^23^
Cd1	0.02423 (18)	0.02423 (18)	0.0238 (3)	0.000	0.000	0.000
Cd2	0.02750 (19)	0.02750 (19)	0.0201 (3)	0.000	0.000	0.000
Br1	0.0365 (3)	0.0530 (3)	0.0555 (3)	−0.0060 (2)	−0.0071 (2)	0.0323 (3)
Br2	0.0464 (3)	0.0296 (2)	0.0354 (3)	−0.00354 (19)	0.0193 (2)	−0.00600 (19)
N1	0.048 (3)	0.055 (3)	0.051 (3)	−0.016 (2)	0.014 (2)	−0.005 (2)
N2	0.056 (3)	0.050 (3)	0.045 (3)	−0.008 (2)	0.013 (2)	−0.017 (2)
C1	0.053 (3)	0.041 (3)	0.045 (3)	−0.010 (2)	0.024 (3)	−0.009 (2)
C2	0.052 (4)	0.046 (3)	0.060 (4)	−0.010 (3)	0.008 (3)	−0.003 (3)
C3	0.060 (4)	0.039 (3)	0.051 (4)	−0.009 (2)	0.009 (3)	−0.015 (3)
C4	0.060 (4)	0.079 (5)	0.079 (5)	−0.039 (4)	−0.013 (4)	0.020 (4)
C5	0.135 (8)	0.054 (4)	0.082 (6)	0.022 (4)	−0.028 (5)	0.001 (4)
C6	0.065	0.054	0.079	−0.017	−0.022	0.008
C7	0.081	0.059	0.115	−0.022	−0.051	0.046

### Geometric parameters (Å, º)

**Table:**

Cd1—Br1^i^	2.5745 (6)	C4—H42	0.9596 (11)
Cd1—Br1^ii^	2.5745 (6)	C4—H43	0.9633 (8)
Cd1—Br1	2.5745 (6)	C4—H4A	0.9691 (8)
Cd1—Br1^iii^	2.5745 (6)	C4—H4B	0.9666 (8)
Cd2—Br2^iv^	2.5806 (5)	C5—C7	1.350 (11)
Cd2—Br2^v^	2.5806 (5)	C5—H51	0.9681 (8)
Cd2—Br2	2.5806 (5)	C5—H52	0.9543 (10)
Cd2—Br2^vi^	2.5806 (5)	C5—H53	0.9706 (9)
N1—C1	1.356 (8)	C5—H5A	0.9700 (8)
N1—C2	1.360 (7)	C5—H5B	0.9552 (8)
N1—C4	1.450 (6)	C6—H42	0.488 (16)
N2—C1	1.297 (7)	C6—H61	0.9600
N2—C3	1.359 (8)	C6—H62	0.9600
N2—C5	1.421 (6)	C6—H63	0.9600
C1—H1	0.9300	C7—H52	0.987 (16)
C2—C3	1.373 (9)	C7—H53	1.241 (15)
C2—H2	0.9300	C7—H71	0.9600
C3—H3	0.9300	C7—H72	0.9600
C4—C6	1.355 (18)	C7—H73	0.9600
C4—H41	0.9610 (8)		
			
Br1^i^—Cd1—Br1^ii^	109.14 (3)	H4A—C4—H4B	106.88 (8)
Br1^i^—Cd1—Br1	109.638 (17)	C7—C5—N2	126.5 (5)
Br1^ii^—Cd1—Br1	109.638 (17)	C7—C5—H51	123.3 (5)
Br1^i^—Cd1—Br1^iii^	109.638 (17)	N2—C5—H51	109.8 (2)
Br1^ii^—Cd1—Br1^iii^	109.638 (17)	C7—C5—H52	46.9 (8)
Br1—Cd1—Br1^iii^	109.14 (3)	N2—C5—H52	111.0 (2)
Br2^iv^—Cd2—Br2^v^	112.71 (3)	H51—C5—H52	109.27 (10)
Br2^iv^—Cd2—Br2	107.878 (14)	C7—C5—H53	62.1 (8)
Br2^v^—Cd2—Br2	107.878 (14)	N2—C5—H53	109.7 (2)
Br2^iv^—Cd2—Br2^vi^	107.878 (14)	H51—C5—H53	107.93 (9)
Br2^v^—Cd2—Br2^vi^	107.878 (14)	H52—C5—H53	109.06 (12)
Br2—Cd2—Br2^vi^	112.71 (3)	C7—C5—H5A	107.1 (7)
C1—N1—C2	108.3 (5)	N2—C5—H5A	105.9 (2)
C1—N1—C4	126.1 (4)	H51—C5—H5A	43.85 (3)
C2—N1—C4	125.7 (5)	H52—C5—H5A	70.62 (6)
C1—N2—C3	110.2 (6)	H53—C5—H5A	141.27 (15)
C1—N2—C5	124.7 (5)	C7—C5—H5B	102.3 (8)
C3—N2—C5	125.1 (5)	N2—C5—H5B	106.7 (2)
N2—C1—N1	108.3 (5)	H51—C5—H5B	64.21 (5)
N2—C1—H1	125.8	H52—C5—H5B	141.30 (18)
N1—C1—H1	125.8	H53—C5—H5B	47.87 (4)
N1—C2—C3	106.6 (6)	H5A—C5—H5B	107.23 (8)
N1—C2—H2	126.7	C4—C6—H42	29.2 (17)
C3—C2—H2	126.7	C4—C6—H61	109.5
N2—C3—C2	106.6 (5)	H42—C6—H61	136.1
N2—C3—H3	126.7	C4—C6—H62	109.5
C2—C3—H3	126.7	H42—C6—H62	86.2
C6—C4—N1	123.2 (8)	H61—C6—H62	109.5
C6—C4—H41	105.9 (7)	C4—C6—H63	109.5
N1—C4—H41	109.7 (2)	H42—C6—H63	102.6
C6—C4—H42	14.4 (8)	H61—C6—H63	109.5
N1—C4—H42	109.7 (2)	H62—C6—H63	109.5
H41—C4—H42	109.44 (11)	C5—C7—H52	44.9 (4)
C6—C4—H43	98.2 (8)	C5—C7—H53	43.7 (4)
N1—C4—H43	109.6 (2)	H52—C7—H53	88.7 (8)
H41—C4—H43	109.13 (9)	C5—C7—H71	109.5
H42—C4—H43	109.23 (11)	H52—C7—H71	98.7
C6—C4—H4A	106.1 (7)	H53—C7—H71	108.7
N1—C4—H4A	106.8 (2)	C5—C7—H72	109.5
H41—C4—H4A	3.993 (3)	H52—C7—H72	72.9
H42—C4—H4A	108.60 (11)	H53—C7—H72	139.5
H43—C4—H4A	112.92 (9)	H71—C7—H72	109.5
C6—C4—H4B	106.0 (8)	C5—C7—H73	109.5
N1—C4—H4B	106.9 (2)	H52—C7—H73	148.2
H41—C4—H4B	103.25 (8)	H53—C7—H73	68.9
H42—C4—H4B	117.45 (12)	H71—C7—H73	109.5
H43—C4—H4B	8.460 (7)	H72—C7—H73	109.5
			
C3—N2—C1—N1	−0.2 (6)	C5—N2—C3—C2	−179.7 (4)
C5—N2—C1—N1	179.1 (4)	N1—C2—C3—N2	0.8 (6)
C2—N1—C1—N2	0.7 (6)	C1—N1—C4—C6	51.2 (10)
C4—N1—C1—N2	−178.3 (4)	C2—N1—C4—C6	−127.6 (10)
C1—N1—C2—C3	−0.9 (6)	C1—N2—C5—C7	10.0 (12)
C4—N1—C2—C3	178.0 (4)	C3—N2—C5—C7	−170.9 (11)
C1—N2—C3—C2	−0.4 (6)		

Symmetry codes: (i) *y*−1/4, −*x*+1/4, −*z*+1/4; (ii) −*y*+1/4, *x*+1/4, −*z*+1/4; (iii) −*x*, −*y*+1/2, *z*; (iv) −*y*+3/4, *x*+3/4, −*z*+3/4; (v) *y*−3/4, −*x*+3/4, −*z*+3/4; (vi) −*x*, −*y*+3/2, *z*.

### Hydrogen-bond geometry (Å, º)

**Table:**

*D*—H···*A*	*D*—H	H···*A*	*D*···*A*	*D*—H···*A*
C1—H1···Br2^vii^	0.93	2.77	3.679 (6)	167
C2—H2···Br1^viii^	0.93	2.93	3.824 (7)	161
C3—H3···Br1	0.93	2.90	3.753 (6)	154

Symmetry codes: (vii) −*x*+1/2, −*y*+3/2, −*z*+1/2; (viii) −*y*+3/4, *x*+1/4, *z*+1/4.
